# Is T Cell Negative Selection a Learning Algorithm?

**DOI:** 10.3390/cells9030690

**Published:** 2020-03-11

**Authors:** Inge M. N. Wortel, Can Keşmir, Rob J. de Boer, Judith N. Mandl, Johannes Textor

**Affiliations:** 1 Department of Tumor Immunology, Radboud Institute for Molecular Life Sciences, Geert Grooteplein 26-28, 6525 GA Nijmegen, The Netherlands; 2 Theoretical Biology, Department of Biology, Utrecht University, Padualaan 8, 3584 CH Utrecht, The Netherlands; c.kesmir@uu.nl (C.K.); r.j.deboer@uu.nl (R.J.d.B.); 3 Department of Physiology, McGill University, 3649 Promenade Sir William Osler, Montreal, QC H3G 0B1, Canada; Judith.mandl@mcgill.ca

**Keywords:** negative selection, central tolerance, self-nonself discrimination, T cell repertoires, artificial immune system, learning by example

## Abstract

Our immune system can destroy most cells in our body, an ability that needs to be tightly controlled. To prevent autoimmunity, the thymic medulla exposes developing T cells to normal “self” peptides and prevents any responders from entering the bloodstream. However, a substantial number of self-reactive T cells nevertheless reaches the periphery, implying that T cells do not encounter all self peptides during this negative selection process. It is unclear if T cells can still discriminate foreign peptides from self peptides they haven’t encountered during negative selection. We use an “artificial immune system”—a machine learning model of the T cell repertoire—to investigate how negative selection could alter the recognition of self peptides that are absent from the thymus. Our model reveals a surprising new role for T cell cross-reactivity in this context: moderate T cell cross-reactivity should skew the post-selection repertoire towards peptides that differ systematically from self. Moreover, even some self-like foreign peptides can be distinguished provided that the peptides presented in the thymus are not too similar to each other. Thus, our model predicts that negative selection on a well-chosen subset of self peptides would generate a repertoire that tolerates even “unseen” self peptides better than foreign peptides. This effect would resemble a “generalization” process as it is found in learning systems. We discuss potential experimental approaches to test our theory.

## 1. Introduction

To eliminate pathogens without damaging healthy cells, the immune system must discriminate between self and foreign (nonself). The innate arm of the immune system does so to some extent using a limited number of germline-encoded receptors that recognize pathogen-associated molecular patterns. By contrast, the adaptive arm of the immune system, which is found in all jawed vertebrates and is mediated by T and B lymphocytes, uses a vastly diverse repertoire of receptors to generate specific protective responses against any pathogen it encounters [1,2]. For example, humans have a repertoire of at least 10^7^ different T cells [3], each expressing one or two of the >10^15^ unique receptor sequences that can arise from the stochastic recombination of V(D)J gene segments and addition of non-templated nucleotides [4,5]. These T cell receptors (TCRs) recognize short foreign peptides presented on major histocompatibility complex (MHC) molecules on the surface of infected or cancerous cells.

The random TCR generation process is required to achieve this diversity, but it inevitably also produces TCRs that recognize self peptides presented by healthy cells. It was long thought that these self-reactive receptors are effectively eliminated during T cell development in the thymus through a process termed negative selection [6]. However, current estimates of how many self peptides each T cell encounters in the thymus range from $10^{3}$ to $10^{5}$ [7,8,9], at least one order of magnitude lower than the total number of possible self peptides. Indeed, recent studies have found that self-reactive T cells are abundant in the periphery after all, especially in humans [10,11,12].

**Figure 1:**
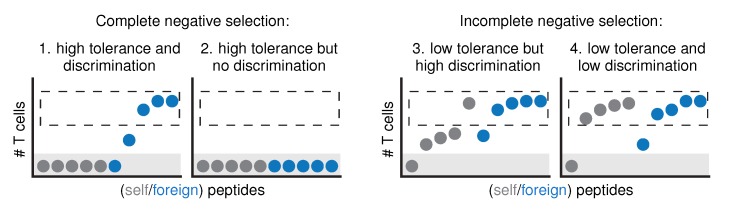
The relationship between tolerance and discrimination becomes more complex when negative selection is incomplete. If negative selection were “complete”, all self peptides would be presented in the thymus and all self-reactive T cells would be silenced (case 1). In other words, all self peptides would be completely *tolerated* (no responding T cells left, gray area), and there would be perfect self-foreign *discrimination* (dashed region: all peptides that are still properly recognized are foreign). The only way to have no discrimination in this scenario is if negative selection would be “too complete”, such that not only all self peptides, but also all foreign peptides are completely tolerated (case 2). If negative selection is incomplete, low tolerance can occur with either very strong (case 3), or very low discrimination (case 4).

This confirmation that negative selection is far from complete has important implications for the relationship between self *tolerance* and self-foreign *discrimination* (Figure 1). When negative selection is “complete” and removes all self-reactive T cells, self-foreign discrimination is simply a consequence of achieving tolerance (Figure 1, case 1). There is one exception to this rule [10,13]: when the selection process removes so many T cells that “holes” arise in the repertoire, some pathogens are no longer detected either and we cannot speak of discrimination anymore—even if there is tolerance (Figure 1, case 2). Incomplete negative selection means that the relationship between tolerance and discrimination becomes less straightforward: selection on a subset of self peptides will likely achieve only low tolerance in itself, but the resulting discrimination can range from very low to very high values (Figure 1, cases 3 and 4). Which of these scenarios applies to our immune system then depends on the question: can negative selection give our T cell repertoire the ability to differentiate between foreign peptides and self peptides they *haven’t seen* in the thymus?

Many learning systems tasked with inferring a *concept* can do so based on a set of *examples*. For example, children infer the concept of English grammar from example sentences they hear and can then construct other sentences they have not heard before. This effect is called *generalization* [14,15], and it does not require the set of examples to cover the complete concept. Here, we hypothesize that a similar generalization effect might occur as a result of T cell negative selection. If this were the case, it could compensate for the incomplete set of self peptides in the thymus. Negatively selected T cell repertoires could then respond differently to self peptides not encountered in the thymus than to foreign peptides, even when selection has little impact on tolerance (Figure 1, case 3). In summary, we ask: can the T cell repertoire “learn by example” during negative selection?

We approach this central question in two steps. First, we ask: can the process of negative selection cause learning by example *in principle*, and if so, under which conditions can this occur? To answer this question, we investigate how a computer algorithm based on a negative selection procedure [16] solves a basic, well-interpretable classification problem outside of immunology: distinguishing English from other languages based on short *strings* (letter sequences) of text. This problem mimics the task of self-foreign discrimination because, in both cases, classes (languages or proteomes) are to be distinguished based on a limited amount of information (short strings or peptides) from only the “self” class. In addition to this analogy, the language classification problem has several useful properties: (1) it is intuitive to understand, (2) it can take on a range of difficulties depending on the languages to be compared [17]; and, (3) since we already know this problem can be solved through generalization by other algorithms [17], it is well-suited for a proof of concept that negative selection can do the same. Using a computational model of negative selection on strings from different languages, we will show that negative selection can indeed allow language discrimination as long as certain conditions are met.

Second, based on the insights gained in this first part, we ask: are these conditions fulfilled when we consider self-foreign discrimination by T cells? By modifying our model such that it recognizes real peptide sequences from the human proteome and various pathogens, we show that the task faced by our immune system is relatively difficult because self and foreign peptides can be very similar to each other. However, we also show that this difficulty can be overcome if the peptides used for negative selection are chosen in a “smart” way that reduces redundancy.

## 2. Results

### 2.1. Problem Definition and Model Design

Throughout this paper, we consider the problem of self-foreign discrimination defined as follows: after negative selection on only a subset of all self peptides (“seen self”), T cells are exposed to both “unseen” self peptides and foreign peptides, and the response against both is measured. Discrimination occurs when the repertoire responds more strongly to the foreign peptides than to the unseen self peptides. In particular, we will focus on discrimination among the peptides recognized by the most TCRs: given that these tend to elicit stronger immune responses [18], the risk of detrimental effects is much higher if self and foreign are confused among these peptides. Assessing discrimination then depends on how we define the “response” to a given peptide. Here, we consider all T cells reacting to a peptide to be important—regardless of their exact affinities. This choice was motivated by evidence that both low and high affinity TCRs are important contributors to immune responses [19].

To investigate under which conditions negative selection can accomplish such discrimination in a T cell repertoire, we use an “artificial immune system” (AIS) [20]. Our AIS is an algorithmic model of a T cell repertoire [16], similar to how an artificial neural network (ANN) is an algorithmic model of the central nervous system. Like ANNs, AISs are not only used for in silico modelling of the biological system, but are in fact general-purpose classification algorithms that can process almost arbitrary input data. This generality of AISs will allow us to use the *same algorithm* to investigate both the original self-foreign discrimination problem and its language classification analogy.

**Figure 2:**
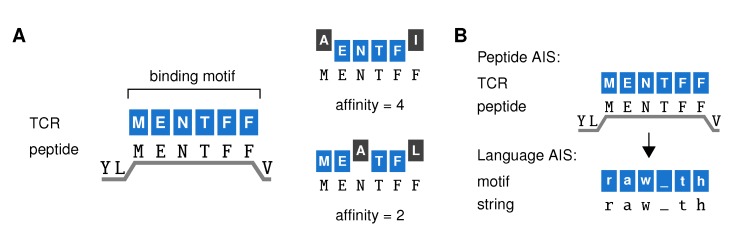
An artificial immune system model of a T cell repertoire. (**A**) Our artificial immune system (AIS) represents TCRs by a *binding motif*—the peptide sequence they bind to most strongly (left). Since TCR binding to peptides on MHC-I (HLA-A2:01) focuses on the six residues at positions 3–8 of the peptide, TCRs are represented as 6-AA sequences. Their affinity for any given peptide equals the maximum number of adjacent positions where the TCR binding motif matches the peptide (right). (**B**) This AIS model can be adapted to distinguish *strings* from different languages rather than self from foreign peptides. We replace 6-AA peptides with 6-letter strings randomly extracted from books in different languages (which consist of the letters (a–z) and the underscore to represent space and punctuation signs). In the language AIS, we speak of general “motifs” rather than “TCRs” to distinguish them from the TCRs in our immune system model.

Our AIS belongs to the family of “string-based” AISs [7,16,21,22] that represents each TCR as a *binding motif*, and defines a motif’s “affinity” for a peptide as the maximum number of adjacent positions where it matches the string (Figure 2A) (detailed methods in Appendix A). We will focus on CD8+ T cells, which recognize peptides bound to the MHC class I (MHC-I) complex with a typical length of nine amino acids (AAs). However, as the six residues at positions 3-8 are thought to be most relevant for TCR binding [23], our TCR motifs also have a length of 6 (Figure 2A). A TCR is then said to *react* to all peptides for which it has an affinity of at least some threshold *t*, which represents a functional response threshold rather than a mere binding threshold. Crucially, reaction does not require a perfect match between the peptide and TCR motif. Thus, our TCRs are “cross-reactive” and react to multiple, related peptides. In contrast to TCR recognition models based on binding energy [24,25], our “motif-based” recognition (Figure 2A) ensures that both peptides recognized by the same TCRs and TCRs recognizing the same peptide share sequence elements—in line with observations from TCR-specific peptide sets [26,27,28] and peptide-specific TCR repertoires [29,30]. Because it was important to consider systems of realistic scale and complexity, we exploited data compression techniques that allow building AISs containing billions of TCRs [22].

Having defined this model, we apply the same principle to build an AIS that distinguishes English from other languages based on short strings of text (Figure 2B). Replacing the six central residues of the peptides by 6-letter strings, we can construct motifs in the same way as before (we will call these “motifs” to distinguish them from the real “TCRs” in the peptide AIS). Although as little as three to four letters can suffice to identify languages in many cases [31], here we chose to use 6-letter sequences analogous to the TCR-peptide model. In Section 2.2 and Section 2.3, we will now first switch to this language AIS to examine whether negative selection can lead to generalization in principle.

### 2.2. An Artificial Immune System Discriminates Self from Foreign after Negative Selection

**Figure 3:**
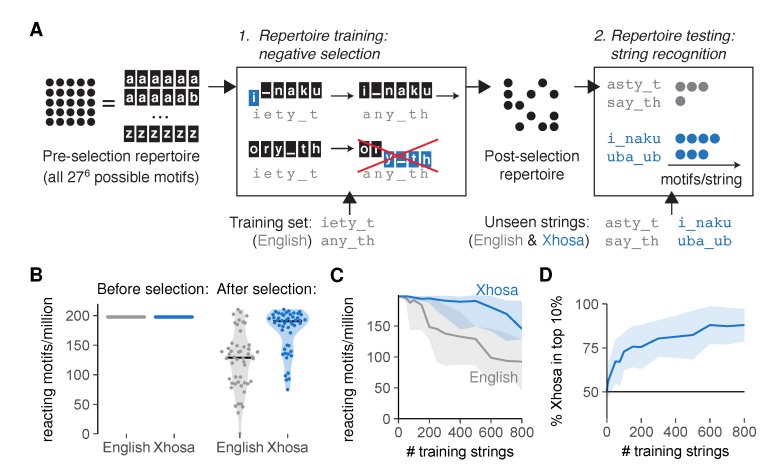
An artificial immune system tasked with language recognition discriminates self and foreign after negative selection on a subset of self. (**A**) Simulating negative selection in silico: (1) Motifs in the unbiased pre-selection repertoire (with all possible 27^6^ ≈ 400 million motifs of six characters (a–z and _)) are deleted if their affinity for any of the *training strings* exceeds the functional response threshold *t*. (2) Unseen English and Xhosa strings are exposed to the post-selection repertoire to find the number of remaining motifs reacting to them with affinity ≥ *t*; (**B**) reacting motifs per million for unseen English and Xhosa strings, before and after negative selection on 500 English strings (∼1 page of text). Horizontal lines indicate medians. Each dot represents a test string, all from a single simulation; (**C**) median and interquartile range of English- and Xhosa-reactivity after negative selection on English strings, obtained from one simulation per training set size; (**D**) percentage of Xhosa strings among the 10% of strings with the most reacting motifs after negative selection on English strings (mean ± standard deviation, SD, of 30 simulations). No discrimination should result in equal amounts (50%) of English and Xhosa strings in this top 10%. Throughout this figure, we tested 50 English and 50 Xhosa strings using an affinity threshold *t* = 3 for negative selection.

The language classification problem can take on a range of difficulties [17], as very dissimilar languages such as English and the South-African language Xhosa are much easier to distinguish than related languages such as modern and medieval English. For a proof of principle that negative selection can allow language discrimination, we first considered the “easy” problem of distinguishing two very dissimilar languages. To test how well our AIS could discriminate between English and Xhosa after incomplete negative selection, we started with an unbiased pre-selection repertoire with equal numbers of motifs reacting to English and Xhosa, and then performed in silico negative selection on an English *training set* by deleting all motifs reacting to any of the (<1000) training strings (Figure 3A, using a threshold *t* = 3 leading to intermediate cross-reactivity). Although this negative selection did not completely abrogate reactivity towards English strings outside of the training set, it still biased the post-selection repertoire to contain more motifs reacting to Xhosa than to English (Figure 3B,C). The 10% most frequently recognized strings in our simulation were indeed predominantly Xhosa strings (Figure 3D and Figure S1A). The affinity distribution of these interactions was shifted towards higher affinities for Xhosa, but only very slightly (Figure S1B)—supporting our choice to focus on the total number of motifs rather than considering different affinities separately (see Section 2.1).

### 2.3. Discrimination Relies on Moderate Cross-Reactivity and Sequence Dissimilarity

**Figure 4:**
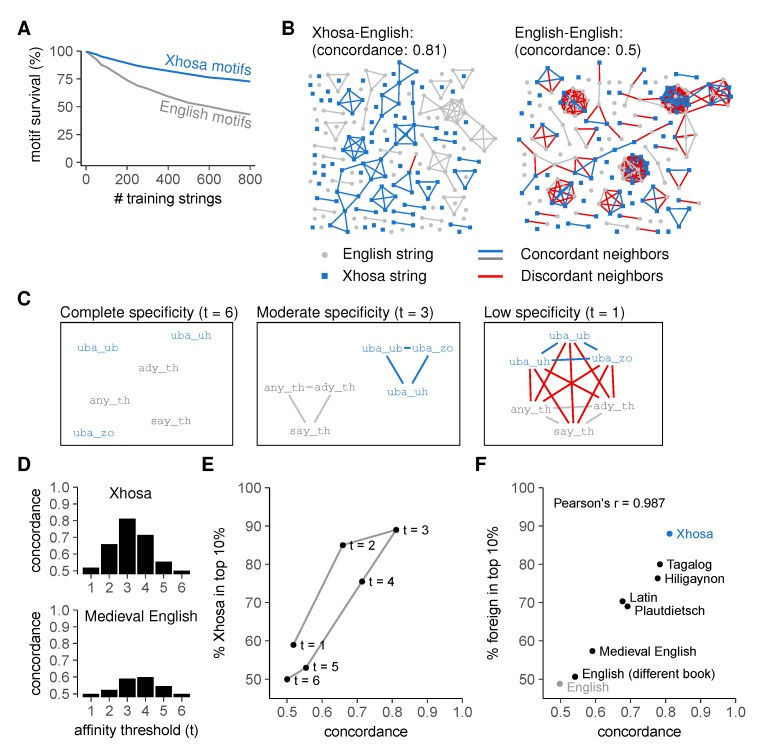
Language discrimination by an artificial immune system requires moderate cross-reactivity and dissimilar self- and foreign strings. (**A**) mean ± standard error of the mean (SEM) percentage of surviving motifs for English and Xhosa strings after negative selection (using threshold *t* = 3). Plot represents a different analysis of data shown in Figure 3C,D; (**B**) string similarity visualized in a graph where nodes (strings) are neighbors (connected by edges) if at least 5/million motifs in the pre-selection repertoire react to both; (**C**) cross-reactivity increases the number of edges between example English and Xhosa strings (demonstrated here for a few examples). Edges between strings from different languages are shown in red; (**D**) concordance in the English-Xhosa and English-Medieval English graphs for different thresholds *t*; (**E**) concordance and discrimination between English and Xhosa for different thresholds *t*. Negative selection was performed on 800 English strings. Datapoint for *t* = 3 corresponds to the endpoint of Figure 3D; (**F**) language concordance versus enrichment of foreign strings among the top 10% most frequently recognized strings after negative selection (*t* = 3, selection on 800 English strings). Pearson’s correlation coefficient r = 0.987, with 95% confidence interval [0.937, 0.997]. The control “English” compares two sets of English strings from the same book that was used for training (Moby Dick), whereas “English (different book)” compares unseen English strings from the training book to those from the Bible. The point "Xhosa" corresponds to the point “*t* = 3” in Figure 4E. See also Figure S1.

These results confirm that our AIS can easily distinguish unseen English from Xhosa even after incomplete negative selection and provide evidence for generalization. To investigate in more detail under which conditions this discrimination arises, we analyzed which motifs were deleted during negative selection on English strings (Figure 4). Motifs reacting to "unseen" English strings—those absent from the "training set" used for negative selection—had a reduced survival compared to motifs reacting to Xhosa strings (Figure 4A). Because motifs are only deleted when they react to at least one string in the training set, this implies that strings eliciting reactions from the same motifs tend to represent the same language. To visualize this, we created graphs in which each node represents a string, and two nodes become connected neighbors when at least five motifs per million pre-selection motifs react to both of them (Figure 4B). Indeed, neighbor strings are largely from the same language (Figure 4B, left), which is quantified by the *concordance*, the average proportion of same-language neighbors. To show that the high concordance (0.81) of English and Xhosa strings represents intrinsic differences between English and Xhosa strings, we randomly divided English strings into two groups and constructed a similar graph, which as expected has a concordance of only 0.5 (Figure 4B, right). This confirms that our AIS only discriminates between sets of strings that are intrinsically different.

Our results indicate two key requirements for achieving self-foreign discrimination through negative selection on an incomplete subset of self: an appropriate level of *cross-reactivity* towards multiple, related strings, and sufficient *dissimilarity* between self-and foreign.

To illustrate the importance of cross-reactivity, we set the affinity threshold in our model to *t* = 6, so that each motif only reacted to the one string that it matches perfectly (i.e., no cross-reactivity). The corresponding graph contains no neighbors at all (Figure 4C, left) and has a concordance of 0.5 (Figure 4D,E). Consequently, lack of cross-reactivity abolishes self-foreign discrimination in our model (Figure 4E) because negative selection cannot delete motifs for strings that are not used for negative selection—it therefore prevents generalization and deletes very few motifs (Figure S1C). Very low specificity (*t* = 1) is equally problematic as it results in a graph where all strings are connected irrespective of language (Figure 4C, right), which leads to low concordance even between dissimilar languages (Figure 4D,E), poor self-foreign discrimination (Figure 4E), and often even deletion of the entire repertoire (Figure S1C). Only intermediate specificities lead to motifs that preferentially react to either English or Xhosa strings (Figure 4C, middle). This results in both a high concordance (Figure 4D,E) and a preference for Xhosa-reactivity in the post-selection repertoire (Figure 4E).

As shown in Figure 4B, even an optimal level of cross-reactivity will not result in a high concordance unless the languages are intrinsically different. The accomplished level of self-foreign discrimination therefore depends directly on the similarity between self- and foreign sequences. Indeed, when we repeated our analysis for a number of other languages with varying similarity to English, we found a linear correlation between concordance and the acquired level of discrimination (Figure 4F). This was a property of the tested languages rather than the specific texts chosen, as our model could not discriminate between English strings from different books (Figure 4F).

In summary, our investigation of the language discrimination problem provided proof of principle that negative selection can lead to a learning effect. It also revealed two requirements for this to happen: (1) the strings to be discriminated must be sufficiently different and (2) cross-reactivity must have an intermediate level. We next asked whether these conditions are met in the real immune system.

### 2.4. Sequence Similarity Hampers Discrimination between Self- and Foreign Peptides

**Figure 5:**
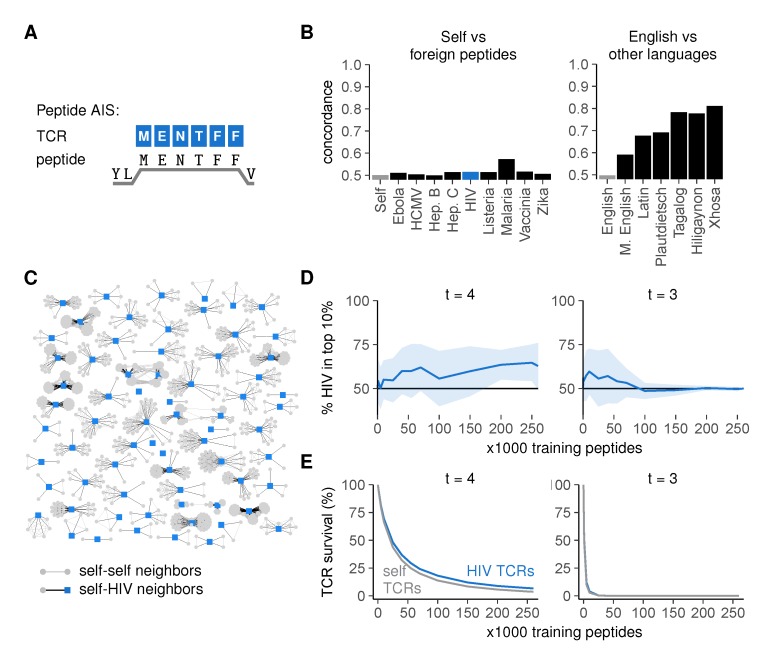
High similarity between self- and foreign peptides hampers their discrimination by the immune system. (**A**) the peptide AIS, in which TCRs bind to peptides on MHC-I (HLA-A2:01) focusing on the six residues at positions 3–8; (**B**) concordance for self versus foreign peptides (left) compared to that for English versus other languages (right). Language concordances from Figure 4F are included for comparison; (**C**) graph of HIV peptides and their neighbors. Edges connect peptides that have at least 5/million pre-selection TCRs in common; (**D**) percentage of HIV-peptides among the 10% most frequently recognized peptides after negative selection (mean ± SD of 30 simulations); (**E**) mean ± SEM percentage surviving TCRs for self and HIV peptides after negative selection.

These results on natural languages suggest that TCR cross-reactivity and sequence dissimilarity should also be important for self-foreign discrimination in the immune system. We therefore returned to our AIS model of self-foreign discrimination by CD8+ T cells (Section 2.1, Figure 5A). Setting the affinity threshold to an intermediate value of *t* = 4 in this model allowed each TCR to react to roughly one in every 55,000 peptides (Figure S2A)—a cross-reactivity level that reasonably matches an experimental estimate of one in 30,000 [32]. Furthermore, at this level of cross-reactivity, peptides elicited reactions from 0 to 20 TCRs per million in our simulated repertoires (Figure S2B), in line with experimental data [33,34,35,36]. These results suggest that the cross-reactivity level of TCRs roughly matches that of our model at *t* = 4, well within the "moderate" range allowing discrimination between dissimilar strings (Figure 4D,E).

To examine whether self- and foreign peptides are dissimilar enough to allow self-foreign discrimination, we first predicted MHC-I-binding peptides from the human proteome [37] and used the residues 3–8 as MHC-bound self peptides in our model. To obtain foreign sequences, we predicted MHC binders for a variety of pathogens associated with T cell immunity: the malaria parasite, the bacterium *Listeria monocytogenes*, and the viruses ebola, hepatitis B, hepatitis C, human cytomegalovirus (HCMV), human immunodeficiency virus (HIV), and vaccinia (Table A1 in Appendix A).

Graphs of self versus foreign peptides had strikingly low concordances (Figure 5B) (detailed methods in Appendix A), barely exceeding the control concordance observed between two random, different sets of self peptides (“Self”, negative control), much lower than, for instance, the concordance we had observed between modern and medieval English. This was a property of the sequences themselves rather than the chosen threshold *t* (Figure S3A). In a graph of all HIV peptides and their neighbors, the majority of HIV peptides had many self neighbors, whereas none of them had HIV neighbors (Figure 5C)—indicating that most HIV peptides are more similar to peptides from the human proteome than to other HIV peptides.

This high similarity between self- and foreign peptides suggests that achieving self-foreign discrimination via negative selection is difficult. To test this, we determined how well a TCR repertoire model could distinguish seen from unseen pathogenic peptides after negative selection on subsets of the human self. Indeed, although the realistic cross-reactivity at *t* = 4 allowed some discrimination between self- and HIV peptides as shown by a small enrichment of HIV among most frequently recognized peptides (Figure 5D and Figure S2C, left), this effect was small even with large numbers of training self peptides. Consistent with this observation, the survival of self-reactive TCRs was only slightly lower than that of HIV-reactive TCRs (Figure 5E, left). These results were not specific for HIV peptides, as we obtained similarly low levels of self-foreign discrimination for all other pathogens tested (Figure S3B). Self-HIV discrimination was even worse for *t* = 3 and rapidly disappeared completely as TCR survival diminished for large training sets (Figure 5D,E and Figure S2C, right), confirming that self-foreign discrimination becomes more difficult when TCRs are too cross-reactive.

### 2.5. Selection on Non-Random Peptides Greatly Improves Self-Foreign Discrimination

Thus, although incomplete negative selection can achieve self-foreign discrimination in principle, achieving sufficient discrimination is very difficult in practice because self- and foreign peptides can be extremely similar and therefore can be recognized by the same TCRs. Clearly, the immune system must overcome this problem in order to balance the removal of self-reactivity with the preservation of foreign recognition. It has previously been suggested that thymic selection should occur on a non-random set of self peptides to achieve self-foreign discrimination [9]. We therefore used our model to investigate what an “optimal” set of self peptides would look like, and how much this might improve self-foreign discrimination.

**Figure 6:**
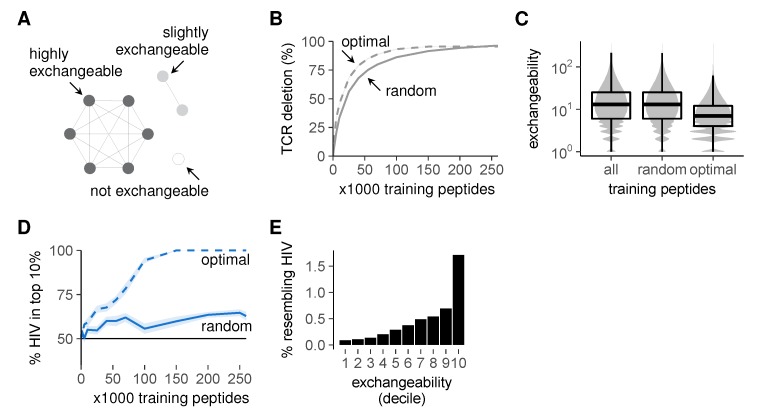
Improved self representation during negative selection allows self-foreign discrimination. (**A**) self peptides from large clusters delete the same TCRs as their neighbors and are thus exchangeable during negative selection, whereas peptides from small clusters are not; (**B**) percentage of self-reactive TCRs deleted by optimal training sets of self peptides during negative selection. TCR deletion with random training sets was computed on the data from Figure 5E for comparison; (**C**) peptide exchangeability distribution in the full set of all self peptides compared to that in random and optimal subsets of 100,000 peptides. Exchangeability is defined as the number of self neighbors + 1; (**D**) self-HIV discrimination after selection on optimal training sets. Discrimination after selection on random training sets (Figure 5D) is shown for comparison. See also Figure S4; (**E**) percentage of self peptides with HIV neighbor(s) plotted against exchangeability (self peptides were divided into 10 equal-number deciles from low to high exchangeability). Negative selection in panels b and d was performed with *t* = 4, and results were plotted as mean ± SEM of 30 simulations.

As a starting point, we based the optimization of the training set on the peptide cluster structure as observed in Figure 5C. The large clusters in this graph contain many similar self peptides, which can delete the same TCRs during negative selection (Figure 6A). Exchanging one such peptide for one of its neighbors during selection thus has little effect on the post-selection repertoire—and presenting both has little added value. By contrast, self peptides in smaller clusters are far less *exchangeable* (Figure 6A): their TCRs cannot be removed as easily by other peptides. Thus, negative selection on randomly chosen training sets is inefficient: these sets often contain several exchangeable peptides that delete the same TCRs, while simultaneously missing many non-exchangeable peptides and allowing the corresponding self-reactive TCRs to escape. We therefore used combinatorial optimization techniques (detailed methods in Appendix A) to compute peptide combinations that deleted as many different self-reactive TCRs as possible (“optimal” training sets, Figure 6B). As expected, these optimal training sets contained fewer exchangeable peptides (Figure 6C, where exchangeability equals the number of self neighbors plus one).

We then tested whether these training sets optimized for inducing *tolerance* could also establish self-foreign *discrimination*. This is not guaranteed, as the latter requires not only the removal of self-reactive TCRs, but also the preservation of foreign-reactivity (Figure 1). Nevertheless, our optimal training sets substantially improved self-foreign discrimination (Figure 6D). This seems to be a consequence of the enrichment for low exchangeability peptides (Figure 6C), which are less likely to delete HIV-reactive TCRs (Figure 6E). Importantly, this discrimination still required appropriate TCR cross-reactivity and was absent at *t* = 3 (Figure S4). From these results, we conclude that negative selection on a representative set of self peptides can alleviate the problem of self-foreign similarity, but only when TCRs are sufficiently specific.

**Figure 7:**
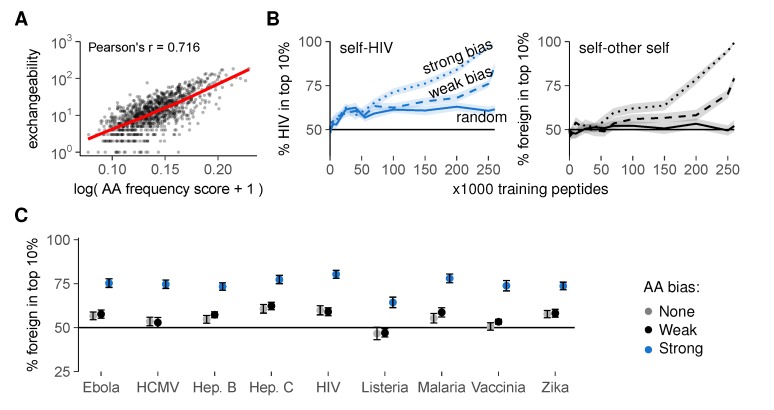
Thymic enrichment for rare AAs facilitates self-foreign discrimination by improving self representation during negative selection. (**A**) exchangeability versus peptide AA frequency score in a random sample of 1000 self peptides (frequency score is low for peptides with many rare AAs (detailed methods in Appendix A)). Pearson’s correlation coefficient r = 0.716, with 95% confidence interval [0.684, 0.745]. See also Figure S5; (**B**) discrimination after negative selection on self peptides chosen with a (weak/strong) bias for rare AAs. Discrimination after selection on random peptides (Figure 5D) is included for comparison. Plots show self-HIV discrimination (left), and self-other self discrimination (right, where a random sample of self was assigned the label “foreign” before selection on training sets from the remaining “self” peptides); (**C**) self-foreign discrimination for different pathogens after negative selection on 150,000 self peptides chosen randomly or with AA bias. See Figure S6 for the full discrimination curves. Negative selection in panels b and c was performed with *t* = 4, and results were plotted as mean ± SEM of 30 simulations.

Obviously, our optimal training sets are artificial, and biological negative selection cannot calculate which self peptides should be present in the thymus. We therefore investigated how a representative set of self peptides might reasonably be obtained during real negative selection. Analysis of our optimal training sets revealed an enrichment for rare AAs compared to the total set of self peptides (Figure S5). Interestingly, peptides with many rare AAs were typically less exchangeable (Figure 7A). This finding suggests that training sets enriched for rare AAs—similar to our optimal sets—contain fewer exchangeable peptides, and might thus result in better self-foreign discrimination.

To test this hypothesis, we again generated training sets of different sizes, but this time picked our training peptides with a probability that depended on the AA composition of each peptide (detailed methods in Appendix A). These probabilities introduced either a weak or a strong bias for self peptides with rare AAs, mimicking the AA enrichment pattern observed in our optimal training sets. This AA bias substantially improved self-foreign discrimination after negative selection, for HIV (Figure 7B, left) and all other pathogens tested (Figure 7C and Figure S6). Interestingly, this strategy also worked when we first set aside a random sample of other self peptides as “foreign” before selecting training sets from the remaining “self” peptides. In this scenario, biased training sets still yielded substantial self-“foreign” discrimination, whereas random sets did not (Figure 7B, right). This result demonstrates that negative selection on non-random training peptides facilitates self-foreign discrimination—even in the extreme case where no inherent difference between self and foreign peptides exists.

## 3. Materials and Methods

### 3.1. Data and Code Availability

All code required to reproduce this paper is available at: http://github.com/ingewortel/negative-selection-2020.

### 3.2. Simulation of Negative Selection

Our general simulation setup can be outlined as follows:

1. Generation of an *unbiased* TCR repertoire containing all possible motifs of length 6. For details, see *Repertoire model of negative selection* (Appendix A.2).
2. Selection of a *training set* of either *n* English strings or *n* self peptides. See *Sequences* (Appendix A.1) for details on the sequences used, and *Training set selection* (Appendix A.3) for details on the manners in which training sets are sampled. The training set selection method was random unless mentioned otherwise in the figure legend. The value of *n* can also be found in the figure legend.
3. Negative selection of TCRs on the training set. All TCR motifs that match *any* of the training sequences in at least *t* adjacent positions are removed from the repertoire. Unless mentioned otherwise, negative selection was performed with an affinity threshold *t* = 3 for strings and *t* = 4 for peptides (see figure legends). All TCRs that remain make up the *post-selection repertoire*. For details on computational methods, see *Repertoire model of negative selection* (Appendix A.2).
4. Analysis of the recognition of *test sequences* by the post-selection repertoire. Test sets always consist of “unseen” sequences that were not part of the training set used for negative selection. See figure legends for details on the number and source of the test sequences used. See *Post-selection repertoire analysis* (Appendix A.5) for details on specific analysis metrics used.

We repeat steps 2–4 with different training and test sets for each simulation. In the case of “optimal” training sets, which are per definition selected only in one way (see *Training set selection* (Appendix A.3) for details), the training set was constant across simulations but the test set was varied. Negative selection success as determined by these simulations is then assessed in the context of expectations based on the similarity between self and foreign sequences (see *Sequence analysis* (Appendix A.4) for details).

### 3.3. Supporting Methods

Detailed computational methods used in this article are available in Appendix A.

## 4. Discussion

In our AIS model, we found that negative selection on an incomplete set of self peptides can bias a T cell repertoire towards foreign recognition. This provides a proof of the principle that, under the right circumstances, negative selection can behave like a learning algorithm: it can let T cell repertoires “learn by example” through generalization. We show that this learning function hinges on two conditions: (1) an appropriate level of cross-reactivity, and (2) sufficient dissimilarity between self and foreign peptides. The basic idea that the immune system acts like a learning system has been pursued within the AIS field for decades [20], but, to our knowledge, our model is the first that investigates such learning using the actual “data” seen by the real immune system: the peptides presented on MHC complexes.

Our results highlight a novel role for T cell cross-reactivity. While it has long been recognized that T cells must be cross-reactive to provide sufficient coverage for the vast number of pathogenic peptides they might encounter [38], our results suggest a second advantage of cross-reactive repertoires: they allow for *generalization*. On the other hand, cross-reactivity should not be too high either: if T cells cannot sufficiently discriminate between peptides, the negatively selected repertoire will *overgeneralize* because (nearly) all T cells will recognize both self and foreign peptides.

This risk of overgeneralization is especially high when self and foreign are highly similar [13,23]. We demonstrate that a non-random subset of self peptides enriched for rare AAs can mitigate this danger by balancing the removal of self-reactive TCRs with the preservation of foreign-reactive receptors. This strategy works even when self and foreign peptides are not inherently different. In fact, for the pathogens we considered, the similarity to self was so high that it is hard to conceive how negative selection on random peptides could achieve any discrimination between foreign and unseen self peptides. By contrast, a “smart” peptide presentation strategy could still ensure that the peptides best recognized by the immune system are predominantly foreign—even in this difficult scenario. This notion would reconcile textbook negative selection theory with recent observations that T cells see only a fraction of all self peptides during thymic selection, and that even healthy individuals have many self-reactive T cells [10].

Although we demonstrate here how negative selection can skew a developing repertoire away from recognition of self, our results also strongly suggest that “central tolerance” by itself cannot achieve reliable self-foreign discrimination. This is in line with the consensus that peripheral tolerance mechanisms are crucial to prevent and dampen immune responses by those self-reactive cells surviving negative selection. Nevertheless—under the right conditions—negative selection can at least provide a *basis* for such other mechanisms to build on. The idea of a “leaky” central tolerance strengthened by peripheral mechanisms is not new [10,39], and is supported for example by studies showing that more nuanced discrimination becomes possible when T cells make decisions cooperatively [40,41]. However, our results clearly show that it is difficult for negative selection to provide even a starting point because it must somehow overcome the fundamental problem of similarity between self- and foreign peptides.

Our finding that non-random peptide presentation improves self-foreign discrimination raises the question how the thymus might obtain a preference for presenting low-exchangeability peptides. Although it remains unclear exactly which and how many peptides a T cell sees during selection, the importance of the thymic peptidome in shaping the TCR repertoire is evident from the existence of specialized antigen presenting cells, transcription factors such as AIRE, and even special proteasomes controlling thymic peptide presentation [42]. We suggest that the biased presentation of low-exchangeability peptides required for self-foreign discrimination might arise from special binding preferences of thymic antigen presentation proteins. As has already been shown for the thymoproteasome during thymic positive selection [43,44], such binding preferences can enrich for specific subsets of self peptides and thereby impact the ability of a TCR repertoire to recognize self and foreign. While a bias for specific AAs such as described in this paper would be one way to enrich for low-exchangeability peptides, we do not exclude that other binding preferences could have a similar impact on self-foreign discrimination.

How could our theory be tested? A first step would be to characterize the peptides present in the thymus during negative selection and to compare these to a hypothetical “random” sample from the proteome. Adamopoulou et al. [45] used peptide elution from dendritic cells in the thymus to identify 842 peptides presented by these cells. It is, however, likely that this dataset is enriched for highly abundant peptides and severely undersamples peptides presented on thymic epithelial cells. These epithelial cells are thought to be the major driver of negative selection, but made up only a small percentage of the cells that were analyzed. More recently, Schuster et al. [46] compiled a nice dataset consisting of MHC class I bound pepdides across different organs. While this dataset is also expected to contain only few peptides from epithelial cells, it could perhaps be used for an initial check whether amino acid distributions of presented peptides differ between the thymus and other organs. However, a key issue with datasets based on mass spectrometry is that this technique itself is biased in the peptides it detects. As such, it currently remains difficult to compare the distribution of eluted peptides to a theoretically predicted reference distribution, which our test would require.

While the discovery of non-random peptide presentation in the thymus would be a first step towards validating our theory, this would still only be indirect evidence based on observational data. A direct proof of our theory would require experimental manipulation of the peptides presented in the thymus. Indeed, the best possible test would perhaps be to choose two different peptide sets with differing amounts of redundancy, and test whether—as predicted by our model—the peptide set with lower redundancy leads to better discrimination of unseen self peptides from foreign peptides. This theoretically ideal test is not yet feasible with currently available experimental techniques. Mice models with only one single peptide present in the thymus have been available for some time [47], and we hope that further development of such experimental models will allow a manipulation-based test of our theory in the future.

At presence, however, the absence of a direct experimental test of our theory remains a major limitation of our work. The exact composition of an “optimal” peptide subset depends on the rules dictating which peptides are recognized by specific T cell receptor sequences, which are still being discovered [29,30], and more knowledge in this area would be required for a firmly testable prediction. However, even though our simple model cannot predict exactly *what* the optimal set of training peptides would be, the finding *that* T cell repertoires can generalize—and that this depends quite strongly on how training peptides are chosen—is independent of the exact model used.

If thymic selection indeed helps self-foreign discrimination by also reducing the recognition of peptides the T cell repertoire has not seen during selection, then this would establish an interesting connection to “slow learning” systems as described in psychology and neuroscience [14,15]. This would show that generalization and “learning by example” in biological systems do not necessarily need to involve neural networks.

## Supplementary Materials

The following are available at https://www.mdpi.com/2073-4409/9/3/690/s1.

## Abbreviations

The following abbreviations are used in this manuscript:

AA	Amino acid
AIS	Artificial immune system
ANN	Artificial neural network
HCMV	Human cytomegalovirus
HIV	Human immunodeficiency virus
MHC	Major histocompatibility complex
SD	Standard deviation
SEM	Standard error of the mean
TCR	T cell receptor

## Appendix A. Supplementary Methods

### Appendix A.1. Sequences

We applied our TCR model to both 6-letter strings and 6-AA peptides. Throughout this methods section, we will refer to them as *strings* and *peptides* for methods specific to either languages or peptides, or as *sequences* for methods applying to both. We will also refer to the motifs in our model as TCRs throughout this methods section. With *self* sequences, we mean either human peptides or English strings, and with *foreign* sequences we mean either pathogenic peptides or strings from other languages (see below).

#### Strings

English training strings (“self”) were extracted from Moby Dick (downloaded from www.gutenberg.org/files/2489/2489.txt). Independent sets of test strings were extracted from translations of the Gospel of John in the Bible (downloaded from www.biblegateway.com). We obtained translations in different languages: English, Medieval English, Latin, and Plautdietsch (Indo-European languages), Tagalog and Hiligaynon (Austronesian languages), and Xhosa (Niger-Congo family of languages). Recognition of these test strings was always compared to recognition of unseen English control strings from the Moby Dick training set. Capital letters were removed and all spaces and punctuation marks were replaced by an underscore (_), yielding text with 27 possible characters (26 letters of the latin alphabet and _). Texts were then randomly cut into strings containing six characters each. Please refer to our code repository (see *Data and code availability* in main text) to obtain the exact input text files with 6-letter chunks.

#### Peptides

Proteomes were obtained from Uniprot [48,49] (Table A1). Potential HLA-A2:01 binders were predicted using NetMHCPan [37] (version 3.0), focusing on peptides of 9 AAs. Using the NetMHCPan default settings, the 2% highest scoring 9-mers were defined as MHC-I binders. Of these, we selected the six residues at positions 3–8 to get the TCR-binding 6-mers, and then removed duplicates to get unique 6-mers for each proteome (Table A1).

**Table A1:** List of proteomes used to extract MHC-I binders. See also *Methods*.

Organism	Proteome Details	Proteins	ID	Download Date (d/m/y)	Unique 6-mers (#)
Ebola virus	Mayinga, Zaire, 1976	9	UP000007209	27/09/2017	140
Human cyto- megalovirus (HCMV)	Human herpesvirus 5 AD169 Isolate Unknown X17403	190	UP000008991	27/09/2017	2090
Hepatitis B virus	Genotype D subtype ayw (isolate France/Tiollais/1979)	7	UP000007930	27/09/2017	65
Hepatitis C virus	H77 isolate Unknown AF009606	2	UP000000518	27/09/2017	112
Human immuno- deficiency virus (HIV)	Type 1 group M subtype B (isolate HXB2)	9	UP000002241	27/09/2017	69
Vaccinia virus	Strain Copenhagen	257	UP000008269	27/09/2017	1955
Zika virus	MR 766 Isolate Unknown AY632535	1	UP000054557	27/09/2017	118
Listeria monocytogenes	serovar 1/2a (strain ATCC BAA-679/EGD-e )	2844	UP000000817	27/09/2017	31,251
Plasmodium ovale (Malaria)	Wallikeri	8636	UP000078550	27/09/2017	89,408
Homo sapiens (human)	-	20,230	UP000005640	01/06/2017	263,216

### Appendix A.2. Repertoire Model of Negative Selection

A limiting factor for simulating negative selection on large TCR repertoires is computational complexity. Our unbiased pre-selection repertoires contain TCRs for every possible binding motif of 6 letters (a–z or _) or 6 AAs—resulting in 27^6^ ≈ 400 million TCRs for the language AIS, and 20^6^ = 64 million TCRs for the peptide AIS. Each of these TCRs needs to be compared against all sequences in the training set. Our implementation of the contiguous affinity model uses advanced computational methods as described in [22,50] to compress T cell repertoires and to enable these comparisons between large sets of sequences. These methods are available in our code repository (see *Data and code availability* in main text).

### Appendix A.3. Training Set Selection

Training sets of *n* English strings were sampled randomly in each simulation. Training sets of *n* self peptides were sampled from the total ∼260,000 human MHC-I binders in one of three ways: random, optimal, or biased sampling (see below for the last two).

#### Optimal Training Peptide Selection

“Optimal” training sets were designed to remove as many self-reactive TCRs as possible. We listed all self-reactive TCR binding motifs that would react to at least one of the ∼260,000 human MHC-I binders for a given threshold *t*, and then selected combinations of minimal numbers of self peptides that would delete a maximal number of these self-reactive TCR motifs. We could not find an exact solution to this combinatorial optimization problem because there is a nearly infinite number of ways to select *n* out of ∼260,000 self peptides—and it is not possible to assess the removal of self-reactive TCRs for each of them. We therefore designed a “greedy” algorithm to find an approximative solution instead. Briefly, we iteratively select the self peptides that remove the most remaining self-reactive TCRs by repeating two steps:

1. List the self-reactive TCR motifs that still remain in the repertoire;
2. Select the self peptide that deletes the most of these remaining self-reactive TCRs. If multiple self peptides delete an equal number of remaining TCRs, we pick only those self peptides that do not overlap in the TCRs they delete.

We stop when all self-reactive TCRs are deleted. The result is an ordered list of self peptides, of which the top *n* epitopes form an “optimal” training set of size *n*. For *t* = 3, an optimally chosen 12,025 self peptides (∼5% of all self peptides) could already remove all self-reactive TCRs, whereas this required 130,407 self peptides (∼50% of all self peptides) at *t* = 4. For simulations with optimal training sets larger than this number, random self peptides were added to the optimal combinations to obtain the desired total number *n*.

#### Biased Training Peptide Selection

To generate training sets biased for rare AAs, all self peptides were first assigned a score that depended on their AA composition:

(A1) $$F_{\mathrm{pep}}=\sum_{p=1}^{6}f_{{}_{\mathrm{aa},p}}$$

with $f_{\mathrm{aa},p}$ the frequency within all self peptides of the AA at position *p* of the 6-mer peptide. These scores were then transformed to a sampling probability $P_{\mathrm{pep}}$ as follows:

(A2) $$P_{\mathrm{pep}}=\frac{\max\left(F\right)-F_{\mathrm{pep}}}{\max(F)-\min(F)}=\frac{6\cdot f_{\mathrm{aa},\max}-F_{\mathrm{pep}}}{6\cdot f_{\mathrm{aa},\max}-6\cdot f_{\mathrm{aa},\min}}$$

where $f_{\mathrm{aa},\max}$ is the frequency of the most common AA (L) in all self peptides, and $f_{\mathrm{aa},\min}$ the frequency of the most rare AA (W). Finally, we sample *n* training peptides from the total set of self peptides using probabilities ($P_{\mathrm{pep}}$)^*s*^, where we use the parameter *s* to control the strength of the bias for rare AAs. Throughout the paper, we used either a weak bias (*s* = 1) or a strong bias (*s* = 5) as indicated in the figures.

### Appendix A.4. Sequence Analysis

#### String Graphs

To visualize strings eliciting reactions from the same TCRs, we constructed a graph where each of 1000 strings from both languages (English and Xhosa or English and more English) was a node. We then counted for each combination of strings how many TCR motifs (pre-selection) could react to both at *t* = 3, and connected their nodes with an edge if this number was at least 10,000.

For visualization, we ordered the connected components (clusters) in this graph by their number of nodes, and plotted every 10th cluster in the final graph.

#### Peptide Graphs

To visualize self and foreign peptides to which the same TCRs react, we again started with a graph with nodes for all self- and foreign peptides, and counted for each pair the number of TCRs that could react to both. This time, we used *t* = 4, and connected peptides with an edge if at least 100 TCRs could react to both.

For visualization of HIV and self peptides, we then selected all connected components (clusters) that contained at least one HIV peptide.

#### Concordance

Concordances were calculated using the full string- and peptide graphs described above (not just the subsets used for visualization). For each node, we listed the proportion of self- and foreign neighbors. If a node was isolated and had no neighbors, we used the expected value $p_{0,\mathrm{class}}$ of this proportion (which equals the proportion of self or foreign nodes in the entire graph). For both the self and foreign class of nodes, we then computed the concordance as the mean proportion $p_{\mathrm{class}}$ of same-class neighbors (so mean proportion of self neighbors for all self nodes, and mean proportion of foreign neighbors for all foreign nodes). Because the ratio between self and foreign peptides/strings was not always equal, we corrected for this ratio as follows:

(A3) $$p_{\mathrm{corr},\mathrm{class}}=\ln\frac{p_{\mathrm{class}}}{1-p_{\mathrm{class}}}-\ln\frac{p_{0,\mathrm{class}}}{1-p_{0,\mathrm{class}}}$$

(A4) $$c_{\mathrm{class}}=\frac{\exp(p_{\mathrm{corr},\mathrm{class}})}{\exp(p_{\mathrm{corr},\mathrm{class}})+1}$$

Here, $p_{0,\mathrm{class}}$ is the expected proportion of same-class neighbors as described above, and $c_{\mathrm{class}}$ is the ratio-corrected mean concordance for that class (self or foreign). This correction ensures that $c_{\mathrm{class}}$ = 0.5 when $p_{\mathrm{class}}=p_{0,\mathrm{class}}$, 0 when there are only discordant edges between nodes of a different class, and 1 when there are only concordant edges between nodes of the same class. To avoid dividing by zero, we set an exception for situations where $p_{\mathrm{class}}=1$:

(A5) $$\mathrm{if}\;p_{\mathrm{class}}==1\to c_{\mathrm{class}}=1$$

The final, total concordance is then computed as a weighted average of the self- and foreign corrected mean concordance:

(A6) $$c=p_{0,\mathrm{self}}\cdot c_{\mathrm{self}}+p_{0,\mathrm{foreign}}\cdot c_{\mathrm{foreign}}$$

#### AA Enrichment

The enrichment of AA *a* ($E_{a}$) was computed as

(A7) $$E_{a}=\ln\frac{f_{a,\mathrm{opt}}}{f_{a,\mathrm{self}}}$$

with $f_{a,\mathrm{opt}}$ the frequency of AA *a* within the optimal set of 130,407 self peptides for *t* = 4 (see *Optimal training peptide selection*), and $f_{a,\mathrm{self}}$ its frequency within the total set of 263,216 self peptides (Table A1).

#### Exchangeability

To compute exchangeability of self peptides, we constructed the graph of all self peptides. We then define exchangeability of a peptide as N+1, where *N* is the number of neighbors in the peptide graph.

To compute how likely peptides of a given exchangeability are to delete foreign-reactive TCRs, we sorted self peptides on their exchangeability and then grouped them into 10 bins with equal numbers of peptides (deciles). Thus, the first decile contains the 10% of peptides with the lowest exchangeabilities, the highest decile the 10% with highest exchangeabilities, etc. We then constructed a graph containing all self and HIV peptides, and analyzed for each decile which percentage of the self peptides in it had an HIV neighbor in this graph (in other words, which percentage “resembled” an HIV peptide).

To analyze the relationship between exchangeability and AA composition, we computed both exchangeability and the AA composition score $F_{\mathrm{pep}}$ (see *Biased training peptide selection*) for 1000 randomly selected self peptides, and analyzed the association between the two scores.

### Appendix A.5. Post-Selection Repertoire Analysis

#### Sequence Recognition

To assess sequence recognition by the post-selection repertoire, we counted the number of post-selection TCRs reacting to each sequence with an affinity of at least the predefined affinity threshold *t* (the same threshold as used for negative selection). Recognition was then reported in the number of reacting TCRs per million TCRs in the post-selection repertoire. If the post-selection repertoire was empty, we set this number to a value of 0. Reported recognition values are always from a single simulation.

#### Self-Foreign Discrimination

To assess self-foreign discrimination within a test set containing equal numbers of self and foreign sequences across multiple simulations, the number of TCRs reacting to each sequence was counted as mentioned above. All sequences were then ranked from high to low numbers of reacting TCRs to obtain the percentage of foreign sequences among the 10% most frequently recognized sequences. When there were ties, we used the value of this percentage that would be expected after random tie-breaking.

#### Affinity Distribution

To compare TCR affinities between strings to which many TCRs react and strings with fewer reacting TCRs, strings were ranked by number of reacting TCRs as described above and split into the top 10% of most-frequently recognized strings and the remaining 90% of strings. For each string, we then counted the number of TCRs reacting to that string with a specific affinity. For both groups, we then computed how many TCRs recognized a string in that group at a given affinity, and report this as a percentage of all TCRs recognizing a string in that group.

#### TCR Survival/Deletion

To assess TCR survival during negative selection on training sets of increasing size, we first chose a test set of self and/or foreign sequences, and listed all pre-selection TCRs whose affinity for these sequences was ≥*t*. We then negatively selected our repertoires on training sets that did not contain any of these test sequences, and assessed the percentage of the TCRs of interest that survived negative selection. TCR deletion can then be computed as 100 minus the TCR survival rate.

### Appendix A.6. Statistical Analysis

Central tendency and spread of asymmetrically distributed continuous variables (sequence recognition in TCRs/million) are described using median and interquartile range. For symmetrically distributed continuous variables (% foreign sequences among 10% most frequently recognized sequences, % TCR survival), we use mean and standard deviation (SD) to show the variability among simulations, or mean and standard error of the mean (SEM) to visualize the uncertainty at the performed number of simulations. Concordances/AA enrichment scores are computed as a single number for a complete set of sequences and therefore have no measure of spread. The Pearson’s correlation coefficient and 95% confidence interval were computed using the cor.test function of the R stats package with default settings (R version 3.3.2, 2016-10-31, RRID:SCR_001905).

We did not perform frequentist statistical testing, since we can generate as many simulation runs as needed to ensure that any interpreted differences are not simply due to random chance. Throughout this paper, we show TCR deletion and self-foreign discrimination curves averaged over 30 simulations. This number of simulations was sufficient that the measurement error—standard error of the mean (SEM)—was small enough in the TCR survival/deletion curves to be invisible. In the self-foreign discrimination curves (% foreign among top 10%), the SEM was not invisible but still small enough with respect to the effect sizes observed (as readers can also judge for themselves in the corresponding graphs).
