# Supplementary material for: Is T Cell Negative Selection a Learning Algorithm?
Source: Cells. 2020 Mar 11;9(3):690. doi: 10.3390/cells9030690 (PMC7140671; doi:10.3390/cells9030690)
Supplement: Supplementary file 1 [file cells-09-00690-s001.pdf]

Supplementary Information

Is T Cell Negative Selection a Learning Algorithm?

Inge M. N. Wortel, Can Keşmir, Rob J. de Boer, Judith N. Mandl, and Johannes Textor

February 29, 2020

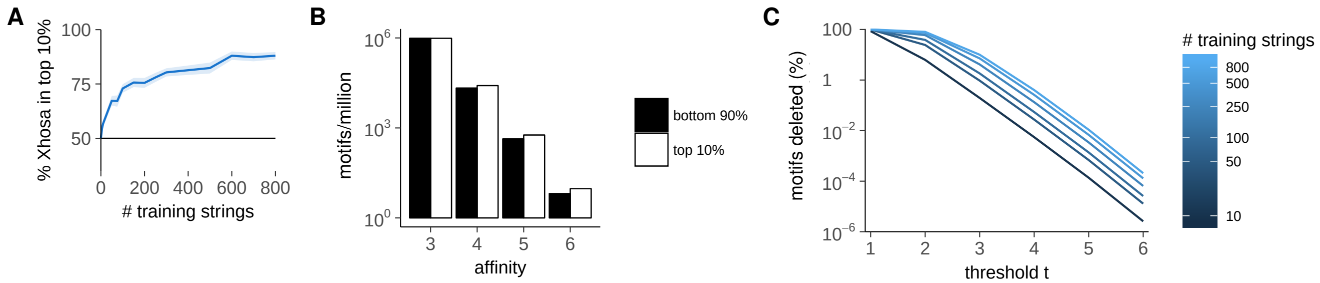

Figure S1: An AIS of string recognition allows simulation of negative selection.
(a) Precision of the discrimination measurement in the data shown in Figure 3D. This plot shows mean±SEM of 30 simulations. (b) Affinity distribution of surviving motifs reacting to 50 English and 50 Xhosa strings after negative selection. Plot shows motif counts (of specified affinity) per million total motifs in either the top 10% of most frequently recognized strings, or the remaining bottom 90% of strings. (c) Average motif deletion rate as a function of the affinity threshold t and the number of training strings used (colored lines). See also Figure 4A, where we plot these data to show motif survival as a function of the training set size at t = 3.

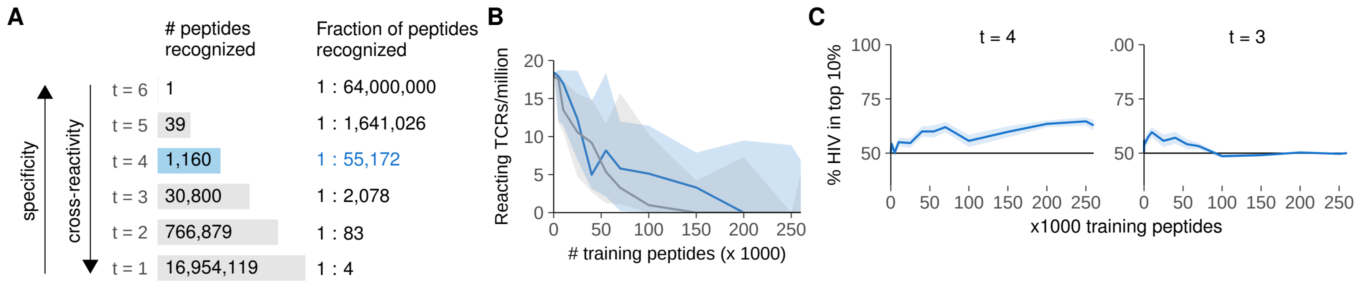

Figure S2: A simple model of TCR-peptide recognition reproduces features of real TCR repertoires.
(a) Cross-reactivity at different affinity thresholds t. At t = 4, a TCR reacts to 1 in every 55,000 peptides, on average.
(b) Reanalysis of the data shown in Figure 5: Typical numbers of TCRs reacting to HIV (blue) and self (gray) peptides after negative selection with t = 4. Plot shows median and interquartile range of reacting TCRS/million. Typical values lie between 0 and 20 TCRs per million, depending on the number of training peptides used for negative selection. (c) Precision of the discrimination measurements in the data shown in Figure 5D. Plots shows mean±SEM of 30 simulations.

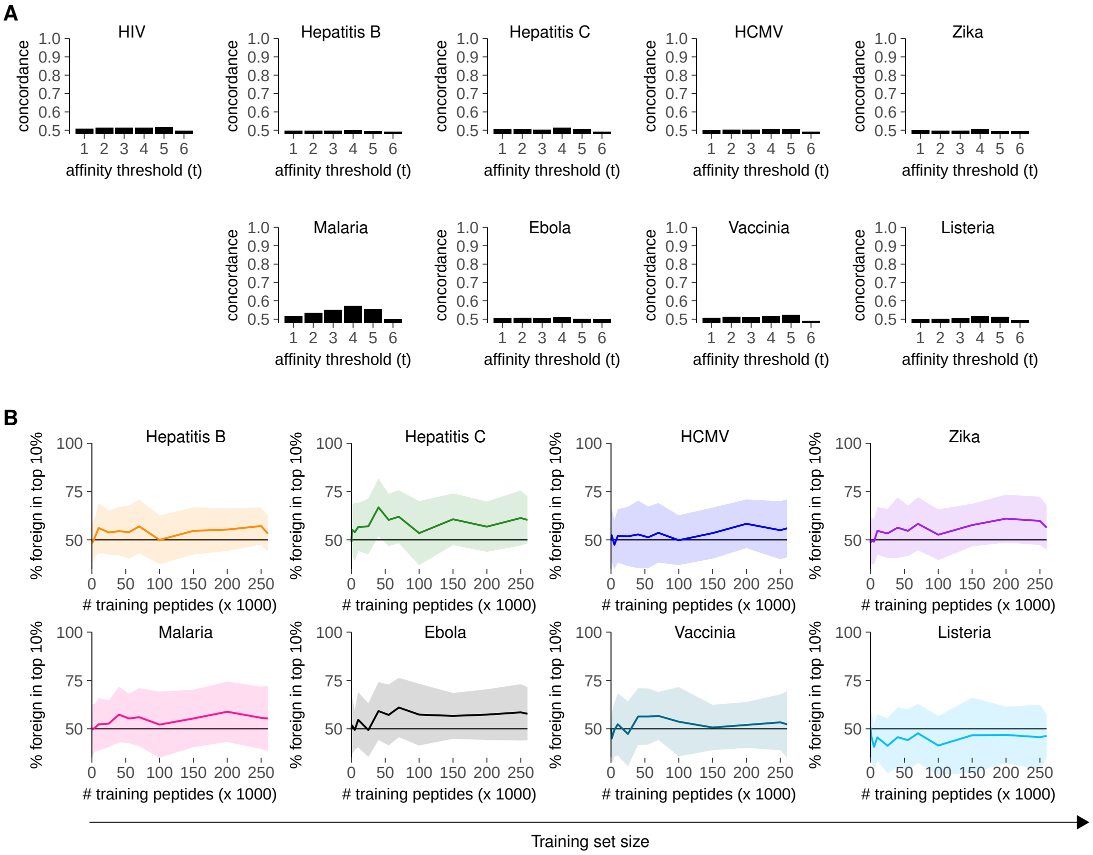

Figure S3: Self-foreign discrimination is poor for all thresholds *t* and all pathogens tested.
(a) Concordance (% of same-class neighbors) in the graph of self and foreign peptides is low for all values of *t* and for all pathogens tested. (b) Self-foreign discrimination after negative selection at $t = 4$ is low for all pathogens tested. Plot show mean$\pm$SD of the percentage foreign peptides among most frequently recognized peptides (30 simulations).

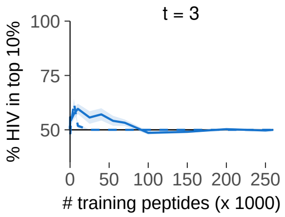

Figure S4: Improved self representation fails to enhance self-foreign discrimination when cross-reactivity is too high.
Plot shows mean±SEM of the percentage HIV peptides among most frequently recognized peptides after negative selection (*t* = 3, 30 simulations). Negative selection was performed on random (solid line, data from Figure 5D included for comparison) or optimal (dashed line) training sets.

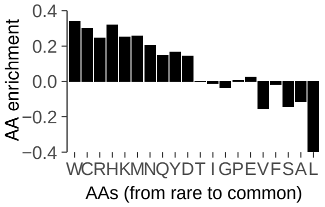

Figure S5: Optimal training sets are enriched for rare AAs.
Plot shows AA enrichment in optimal training set. Enrichment is the log of the observed frequency divided by the frequency among all self peptides. Negative values indicate depletion.

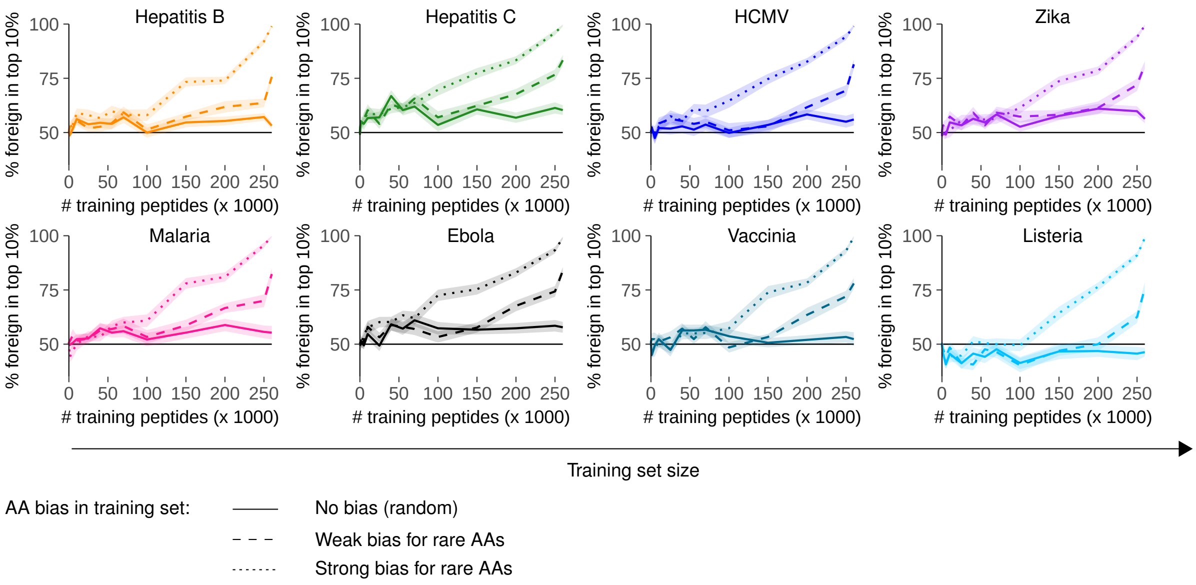

Figure S6: Increased presentation of rare AAs during negative selection improves self-foreign discrimination for all pathogens tested.

Plot shows mean±SEM of the percentage foreign peptides among most frequently recognized peptides after negative selection ($t = 4$, 30 simulations). Training peptides were either chosen randomly (solid line, data from Figure S3B included for comparison) or with a weak/strong bias for peptides with rare AAs (dashed/dotted lines).
